# Diseases of the Abdomen

**Published:** 1902-12-27

**Authors:** 


### DISEASES OF THE ABDOMEN.

The existence of a "quiescent period" in grave abdominal conditions has been recognised before; but Tubby[5] gives some practical points in the diagnosis of such conditions. The difficulty is to know if the improvement in condition is only apparent or real. A patient who has shown alarming symptoms gets apparently much better; these symptoms may, however, be only in abeyance, and a worse condition brewing. This quiescent period is a prominent feature in

(1) Injury to intestines, with or without rupture.

(2) Perforation of intestine by ulceration.

(3) Injury to, and bruising of, the peritoneal connections of the viscera, with interference with their blood supply.

(4) Acute disease of genital organs, especially Fallopian tubes.

(5) Acute appendicitis, either ulcerative, perforative, or gangrenous. It is in this disease that the condition is most often met with.

The clinical signs of moment in these doubtful cases are these :—

(1) Repeated records of pulse and temperature may show a very rapid pulse, while the temperature (surface) is abnormal. This is of grave import, and means septic absorption, but it must be observed early in order to prevent saturation with septic products, in order for operation to be of use.

After an acute attack of appendicitis, when the temperature and pulse rate have been high, if a fall occurs and is followed by a rise in a few days, pus, in quantity, is indicated.

(2) A transient or persistent rigidity is most valuable in the absence of other symptoms, and if peristalsis is absent it is convincing as an indication for immediate interference.

(3) Distension lasting longer than 24 to 36 hours in abdominal conditions is of grave moment and must be relieved.

(4) Vomiting occurring in such a quiescent period in spite of good nursing, careful feeding, and sedatives, must not be underestimated.

(5) Leucocytosis: Counts of over 20,000 in cases of appendicitis indicate pus, gangrene, or peritonitis. Below this number nothing is certain, as in 35 per cent. of cases of appendicitis there is a small increase in leucocytes.

Leucocytosis is absent in typhoid, but present in ovarian abscess, perinephritic suppuration, and, indeed, in suppuration generally. The presence of leucocytosis in cases of gastric ulcer, apart from a certain amount due to severe hæmorrhage or digestion, is an indication of perforation or other grave condition.

In this connection Savill[6] considers that in any grave abdominal trouble the blood count is of the utmost importance, but the presence of pus is to be inferred not by the actual number of the leucocytes as a whole, but from the preponderance over the normal 65 per cent. of the polynuclear cells.

(6) Chest complications persisting when the abdominal symptoms have apparently cleared up indicate exploratory operation for pus in the region of the diaphragm.

In a case giving the symptoms of acute intestinal obstruction, under the care of Tyson and Linington[7] the cause was found to be embolism of branches of the superior mesenteric artery. This had led to gangrene in a large portion of the bowel and the mesentery right up to the attachment. Extensive removal was not sufficient to save the patient's life, death occurring 36 hours later. They remind us that hæmorrhage from the bowel may be due to this cause; extreme fœtor, with tension and tympanitic swelling of the abdomen show gangrenous bowel and peritonitis. Provided operation is performed early enough the prognosis is not necessarily fatal. Kneading the abdomen is the worst treatment that can befall the case; opium masks the symptoms. Should the collateral circulation be established, such conditions may subside by themselves, possibly undiagnosed.

From statistics of Massachusetts General Hospital Shattuck[8] gives the mortality of tubercular peritonitis as 13·2 per cent. This is based upon a series of 98 cases at discharge. The mortality after 2 to 11 years rises to 47·3 per cent.; ultimate mortality under medical treatment, 68 per cent.; under surgical treatment, 37·5 per cent.; two patients have shown recurrence and are now well. The writer concludes from his experience that :—

(1) Patients may recover completely even if the disease be complicated by tubercular disease elsewhere under (*a*) purely medical treatment; (*b*) tapping; (*c*) incision.

2. Best hygienic surroundings are necessary as in other conditions of tubercular disease.

3. We are warranted in trying medical treatment for a time, tapping if necessary, for the relief of discomfort.

4. If no improvement or if loss of ground occur in one month or six weeks of medical treatment, then surgery should be advised.

Bernard[9] draws attention to the resemblance of some cases of lead colic to appendicitis. The diagnosis should be clear, however, in 24 hours. Theodore Fisher[10] gives as his impression that more cases of appendicitis are met with in the post-mortem room, since operative treatment has become more common, than before.

[5] Lancet, May 10. [6] Ibid., [7] Ibid., May 3. [8] Amer. Jour. Med. Sci., July 1902. [9] Brit. Med. Jour., March 1. [10] Lancet, July 19.

(*To be continued.*)

